# LTR retrotransposon landscape in *Medicago truncatula*: more rapid removal than in rice

**DOI:** 10.1186/1471-2164-9-382

**Published:** 2008-08-10

**Authors:** Hao Wang, Jin-Song Liu

**Affiliations:** 1 T-life Research Center, Department of Physics, Fudan University, Shanghai 200433, PR China

## Abstract

**Background:**

Long terminal repeat retrotransposons (LTR elements) are ubiquitous Eukaryotic TEs that transpose through RNA intermediates. Accounting for significant proportion of many plant genomes, LTR elements have been well established as one of the major forces underlying the evolution of plant genome size, structure and function. The accessibility of more than 40% of genomic sequences of the model legume *Medicago truncatula *(*Mt*) has made the comprehensive study of its LTR elements possible.

**Results:**

We use a newly developed tool LTR_FINDER to identify LTR retrotransposons in the *Mt *genome and detect 526 full-length elements as well as a great number of copies related to them. These elements constitute about 9.6% of currently available genomic sequences. They are classified into 85 families of which 64 are reported for the first time. The majority of the LTR retrotransposons belong to either Copia or Gypsy superfamily and the others are categorized as TRIMs or LARDs by their length. We find that the copy-number of Copia-like families is 3 times more than that of Gypsy-like ones but the latter contribute more to the genome. The analysis of PBS and protein-coding domain structure of the LTR families reveals that they tend to use only 4–5 types of tRNAs and many families have quite conservative ORFs besides known TE domains. For several important families, we describe in detail their abundance, conservation, insertion time and structure. We investigate the amplification-deletion pattern of the elements and find that the detectable full-length elements are relatively young and most of them were inserted within the last 0.52 MY. We also estimate that more than ten million bp of the *Mt *genomic sequences have been removed by the deletion of LTR elements and the removal of the full-length structures in *Mt *has been more rapid than in rice.

**Conclusion:**

This report is the first comprehensive description and analysis of LTR retrotransposons in the *Mt *genome. Many important novel LTR families were discovered and their evolution is elucidated. Our results may outline the LTR retrotransposon landscape of the model legume.

## Background

Transposable elements (TEs) are mobile repetitive DNA that have been found in virtually all eukaryotic genomes investigated so far [1-3]. LTR retrotransposons are class I TEs that transpose in a "copy and paste" mode via RNA intermediates. Typical structural characters of a LTR retrotransposon include: 1) two highly similar LTR sequences from several hundred to several thousand bp; 2) 4–6 bp target site duplication (TSD) at its 5' and 3' ends; 3) primer binding site (PBS) downstream of 5' LTR and polypurine tract (PPT) upstream of 3' LTR; 4) protein-coding domains of enzymes important to retrotransposition, e.g. Capsid protein (GAG), Aspartic Proteinase (AP), Reverse Transcriptase (RT), Integrase (IN), and RNase H (RH). Sometimes Envelope protein (ENV) may occur as well [4]. In the plant kingdom, LTR elements present a significant fraction of many genomes and even make predominant components of large genomes [5-7]. The amplification and deletion of these elements is considered to be an important mechanism underlying the remarkable genome size variation in plants [8-11]. Moreover, LTR retrotransposons affect genome organization, gene regulation [12,13], novel gene origination [14,15] and other genetic functions. In summary, the dynamics of LTR retrotransposons are thought to be an important source of genome evolution.

*Medicago truncatula *is a model plant of the Fabaceae, the third largest angiosperm family. Because of their vital role in agriculture and environment [16,17], legumes have provoked great interests. The identification and study of LTR elements is one of the basic and indispensable step to understand biology and evolution of this family. The sequencing of *Mt *opens an unprecedented opportunity to carry out a thorough study of it at the molecular level. Genomic data so far released have made it possible to explore many important facts of the *Mt *genome, specifically, the characteristics of LTR elements and their interactions with the host organism.

In comparison with the Gramineae, the knowledge of LTR retrotransposons in the Fabaceae is relatively limited [18,19]. To date, a few *Mt *LTR families, e.g. MEGY and Ogre have been well documented [20-22] and some families have been deposited in Repbase [23] and TIGR Plant Repeat Databases [24]. However, little research has been focused on the comprehensive identification and description of LTR retrotransposons based on high-throughput *Mt *genomic sequences.

Here we report the result of the computer-based analysis of LTR retrotransposons in 233 Mb *Mt *BAC sequences. At least 85 LTR families were found. We analyzed their phylogenetic relationship and structural patterns, with emphasis on several important families. We investigated the amplification-deletion pattern of these LTR elements and found that the removal of LTR elements in *Mt *has been more rapid than in rice, and more than 10 Mb of LTR retrotransposon sequences have been lost. The present work in about 41% of the whole-genome provides the LTR retrotransposon landscape in *Medicago truncatula*.

## Results and Discussion

### Estimation of copy-number of LTR elements

The development of *ab initio *algorithms [4,25,26] has greatly promoted the identification and analysis of LTR elements in large-scale genomic data. With predicted full-length elements at hand, a widely adopted method to find their related copies is to perform homology search against the host genome. However, subregions of full-length elements may be generated through insertion of other TEs, e.g. nested elements are caused by the insertion of LTR elements one into another [8,9]. In this report, we call these subregions unrelated sequences. Based only on the recognition of structural characters, *ab initio *prediction is not capable to provide information on such sequences. If an element contains unrelated sequences derived from highly abundant TEs, taking the direct matches as its copies will greatly overestimate its copy-number and exaggerate its contribution to the host genome. Therefore, we developed an algorithm (see Methods) to discriminate unrelated sequences and discard the matches generated by them (pseudo-copies). Using this method we obtained a more accurate estimation of the number of *Mt *LTR copies.

### Overview of LTR retrotransposons

This survey identified 526 full-length LTR elements in 232996 Kb *Mt *BAC sequences [see Additional file 1]. The following validation process detected more than 16000 copies, corresponding to 22470 Kb sequences, about 9.6% of all the sequences scanned. If this value is kept in the unreleased genomic sequences, the percentage of the LTR retrotransposons in *Mt *is lower than that in rice (17%–22%) [7,27], yet still remarkably higher than that in *Arabidopsis thaliana *(1–2%) [28]. The length of the full-length elements is within the ranges of 364 bp to 18.7 Kb and that of the LTRs is 126 bp to 3.5 Kb [see Additional file 2]. Most of the elements showed canonical TG-CA boxes and 4–6 bp TSDs.

In this report, We define the LTR family by DNA sequence similarity, following the suggestion of Wicker et al. [3]: two elements belong to the same family if they share 80% (or more) sequence identity in at least 80% of their coding region or internal domain, or within their LTR, or in both. A novel family is discovered when the following standards are met: 1) None of the members in the family belong to the same family with known legume LTR retrotransposons. 2) Besides a full-length member, the family has at least one strong hit (also called strong-hit copies. See Methods).

The 526 full-length elements and their related copies thus were classified into 85 families, of which 64 were identified for the first time. The information of these families is listed in Table 1. LTR families are denoted as MtrXX (XX are digits) and the last two columns of Table 1 list the number of full-length and strong-hit copies because these two values provide multi-copy supports for a family. The following sections sometimes mention the total copy-number of a family and the length of its sequences. Such values, however, are estimated by all copies of that family, including full-length, strong-hit and other truncated ones.

**Table 1:** Summary of the 85 *Medicago truncatula *LTR families

Family	Pre-existing name	Super-family	BAC	Location(bp)	LTR size(bp)	Element size(bp)	FL	Strong hit
Mtr1	-	Copia	AC150246.1	34464–46424	1309	11961	69	184
Mtr2	COPIA4	Copia	AC137553.45	99838–112535	2045	12698	11	29
Mtr3	-	Copia	CR931729.2	71151–77207	1287	6057	5	22
Mtr4	-	Copia	AC157648.18	22659–28446	627	5788	9	15
Mtr5	-	Copia	AC146866.6	121051–127748	602	6698	8	14
Mtr6	-	Copia	AC151725.26	4572–10226	797	5655	10	14
Mtr7	-	Copia	CT967304.4	59158–64899	639	5742	7	13
Mtr8	-	Copia	AC145027.17	30490–37188	676	6699	8	12
Mtr9	SHACOP3	Copia	AC159223.1	27730–32760	189	5031	6	11
Mtr10	-	Copia	AC144482.11	104300–109315	436	5016	10	11
Mtr11	-	Copia	AC165219.2	137152–141889	273	4738	7	10
Mtr12	-	Copia	AC149637.11	7608–18418	1344	10811	5	7
Mtr13	-	Copia	AC151956.5	65260–71649	203	6390	5	6
Mtr14	-	Copia	AC144538.23	111496–121985	1394	10490	5	6
Mtr15	COP20	Copia	AC121235.20	19414–24294	346	4881	5	6
Mtr16	-	Copia	CR931743.1	32761–38060	574	5300	4	6
Mtr17	SHACOP12	Copia	AC148291.22	18569–23612	213	5044	5	5
Mtr18	-	Copia	AC124217.21	65448–73850	1419	8403	2	4
Mtr19	-	Copia	AC149492.14	60016–72184	1822	12169	2	4
Mtr20	-	Copia	AC145330.19	54872–60067	190	5196	3	4
Mtr21	-	Copia	AC144760.27	99316–104667	597	5352	4	4
Mtr22	COP10	Copia	AC144618.7	69511–74634	324	5124	4	4
Mtr23	COP6	Copia	AC125476.30	52976–57511	212	4536	2	4
Mtr24	-	Copia	AC148470.14	69233–73561	250	4329	3	3
Mtr25	SHACOP4	Copia	AC152964.13	48071–52925	276	4855	2	3
Mtr26	-	Copia	CT573053.1	95022–106275	1608	11254	2	3
Mtr27	-	Copia	AC140916.17	54292–65468	1434	11177	1	3
Mtr28	-	Copia	AC174349.10	54241–58572	126	4332	3	3
Mtr29	COP12	Copia	AC165446.16	77640–82389	351	4750	2	3
Mtr30	MTCOPIA2	Copia	AC149471.1	84193–89268	186	5076	2	3
Mtr31	-	Copia	CT573028.11	32334–34027	204	3059	1	3
Mtr32	COP3	Copia	AC138465.22	17893–22580	181	4688	3	3
Mtr33	MTCOPIA1	Copia	CT963078.3	62740–67719	269	4980	2	3
Mtr34	SHACOP11	Copia	AC154867.1	30673–35442	189	4770	1	3
Mtr35	SHACOP20	Copia	AC149038.2	47760–52995	263	5236	2	3
Mtr36	-	Copia	AC174295.7	36459–41224	171	4766	2	2
Mtr37	-	Copia	AC157979.11	69871–74887	249	5017	1	2
Mtr38	-	Copia	AC171778.15	79794–84281	222	4488	2	2
Mtr39	-	Copia	AC148470.14	54043–59356	861	5314	2	2
Mtr40	SHACOP9	Copia	AC175047.2	122584–127374	190	4791	2	2
Mtr41	-	Copia	AC174336.9	98223–102259	296	4704	1	2
Mtr42	-	Copia	AC157506.3	45694–50555	205	4862	1	2
Mtr43	-	Copia	AC175047.2	115144–119648	256	4505	1	2
Mtr44	-	Copia	AC183304.10	99523–104053	167	4531	2	2
Mtr45	-	Copia	CR955009.1	105072–109675	306	4604	2	2
Mtr46	COP14	Copia	AC170583.6	12146–16446	171	4301	2	2
Mtr47	COP21	Copia	AC182817.5	43694–48680	290	4987	2	2
Mtr48	SHACOP21	Copia	AC161106.13	30630–35152	232	4523	2	2
Mtr49	-	Copia	AC158377.1	75346–80903	131	5558	2	2
Mtr50	-	Copia	AC175047.2	83093–87466	242	4374	1	2
Mtr51	-	Copia	AC123573.41	60243–65229	213	4987	1	2
Mtr52	-	Copia	AC135798.31	10206–15241	330	5036	1	2
Mtr53	-	Copia	AC137831.27	31862–36604	218	4782	1	2
Mtr54	-	Copia	CT963132.5	16390–21157	239	5030	1	2
Mtr55	-	Copia	AC149634.8	14602–17988	206	3387	1	2
Mtr56	SHACOP2	Copia	AC146909.23	66018–71021	274	5004	2	2
Mtr57	Ogre1A,B,C,D	Gypsy	AC144405.30	85771–104542	3529	18772	114	137
Mtr58	-	Gypsy	AC162162.23	96896–105667	2127	8772	34	69
Mtr59	Ogre2,3,4	Gypsy	AC138465.22	43789–60338	2435	16550	31	37
Mtr60	-	Gypsy	AC123573.41	34307–46826	1335	12520	2	26
Mtr61	-	Gypsy	AC147430.9	69500–78131	2072	8632	1	10
Mtr62	-	Gypsy	AC160097.27	42464–45517	2076	8661	1	10
Mtr63	-	Gypsy	CU024896.3	74551–82251	2222	7701	3	6
Mtr64	-	Gypsy	AC146759.29	74288–85435	1218	11148	4	5
Mtr65	-	Gypsy	CT963073.3	80019–85667	313	5649	4	5
Mtr66	-	Gypsy	AC150705.16	77136–84299	1943	7164	2	4
Mtr67	-	Gypsy	AC140773.20	23404–37605	721	14202	3	4
Mtr68	-	Gypsy	AC125481.23	24806–33987	330	9182	2	3
Mtr69	-	Gypsy	AC157375.2	85556–93073	2125	7518	3	3
Mtr70	-	Gypsy	AC144591.10	13089–29661	2370	16573	1	3
Mtr71	-	Gypsy	CU024896.3	71754–86395	366	6936	1	2
Mtr72	GYPSHAN2	Gypsy	AC158209.13	42449–47637	409	5189	2	2
Mtr73	-	Gypsy	AC148657.1	69339–74484	394	5146	2	2
Mtr74	-	Gypsy	CU024896.3	86399–92950	819	6552	2	2
Mtr75	-	TRIM	AC147537.35	119057–119409	130	364	9	74
Mtr76	-	TRIM	CR954194.1	12806–16125	652	3320	17	38
Mtr77	-	LARD	AC155884.2	39928–44595	2191	4668	5	26
Mtr78	-	LARD	CT030253.10	41580–46389	921	5497	3	26
Mtr79	-	TRIM	AC158173.13	32092–32698	190	607	5	25
Mtr80	-	env-class	AC146719.32	60449–68395	1239	7947	5	20
Mtr81	-	LARD	CT025840.2	61376–73139	2414	11764	2	14
Mtr82	-	TRIM	CT009479.4	31485–34519	810	3035	7	10
Mtr83	-	LARD	AC140022.11	3262–11660	1440	8399	2	10
Mtr84	-	LARD	AC147201.16	18119–23949	259	5831	6	10
Mtr85	-	TRIM	AC145219.16	111276–112148	249	873	1	6

**Figure 1:**
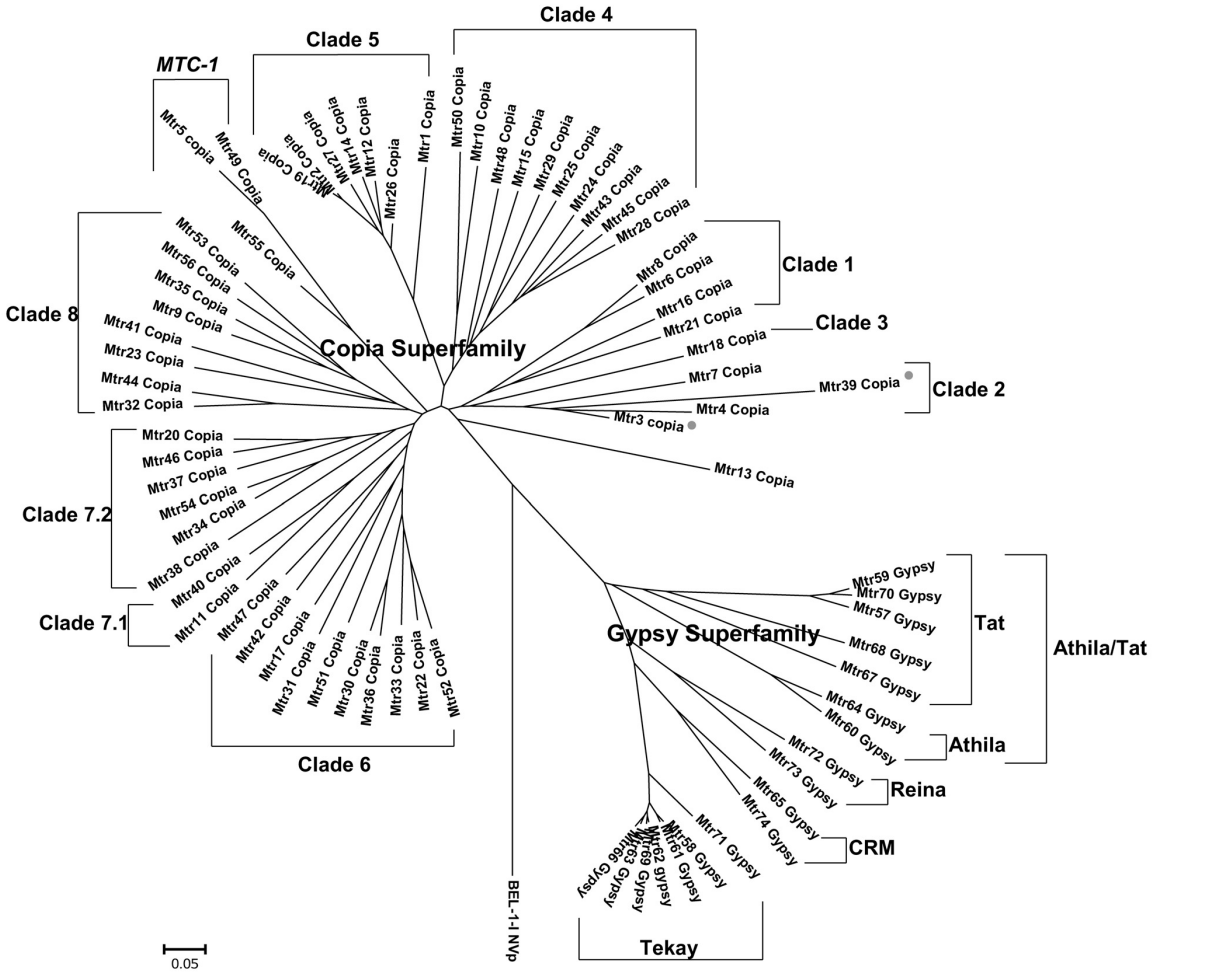
**RT phylogenetic tree of 74 LTR families**. A Bel-Pao type RT (BEL-1-I_NVp from Repbase) is used as outgroup. The 74 families are grouped into Copia or Gypsy superfamily. In the tree, each family is described by its name and a superfamily label. The superfamily label is given according to the order of domains in the POL. RT similarity and domain organization give consistent categorization. Mtr3, Mtr5 and Mtr62 lack other domains except RT, so they are categorized directly though RT similarity and are marked by the lowercase initials of the superfamilies. The 14 clades, to which the 74 families belong, are shown in the figure. The placement of Mtr3 and Mtr39 is unresolved and they are marked by grey dots [see Additional file 4].

Phylogenetic analysis classified 74 families as Copia or Gypsy superfamily (Figure 1) and the rest 11, though quite abundant in genome, could not be categorized as either superfamily by their protein-coding domain organization or sequence similarity. We found that half of the 11 families had long ORFs in their internal domain and some ORFs showed a certain degree of homology with known TE proteins. These families were categorized into TRIMs or LARDs group (TRIMs-LARDs) by their length [3].

### Protein domain organization and phylogenetic relationships within LTR families

We analyzed protein-domain organization of the 85 families (see Methods) and found 9 patterns (Table 2 and [see Additional file 3]). HMMER detected the canonical Copia and Gypsy domain structures, i.e. 5'-GAG-IN-RT-3' and 5'-GAG-RT-IN-3', in 35 and 14 families, respectively. Although it failed to detect the GAG proteins in 21 families, they could still be categorized by the order of their RT and IN in the POL. Mtr3, Mtr5 and Mtr62 had RT but not IN, and they were assigned to either superfamilies by sequence similarity of RT domain (The reason of this assignment is explained in next paragraph): Mtr3 and Mtr5 were categorized as Copia superfamily while Mtr62 as Gypsy superfamily. At last, we obtained 56 Copia-like and 18 Gypsy-like families. Using a RT of Bel-Pao element (BEL-1-I_NVp from Repbase) as outgroup, we constructed the NJ phylogenetic tree of the 74 families based on their RT similarity (Figure 1). The tree branched into two clades and this split was well supported (bootstrap value: 100%). In the tree, the superfamily label of each external node was given according to the order of domains. It is worth noting that the two clades consist of neither more nor less than members of two superfamilies, respectively. In other words, the categorization of superfamily based on RT similarity concurs with that based on domain organization. This means that RT similarity is enough to categorize LTR elements at the level of superfamily. In Figure 1, Mtr3, Mtr5, and Mtr62 are also drawn with others, but the tree topology do not change if they are deleted. These results support their earlier categorization by RT similarity.

**Table 2:** Domain organization of LTR families.

Superfamily	pattern^*a*^	Number of families
Copia	GAG-IN-RT	35
	IN-RT	19
	RT^*b*^	2
Gypsy	GAG-RT-IN	14
	RT-IN	3
	RT^*b*^	1
Other	GAG^*c*^	3
	RT^*d*^	1
	Other	7

^*a *^This table only shows the organization of GAG, IN and RT. See S3.1 for more information.

^*b *^These elements are classified by RT similarity.

^*c *^ORFs inside the three families share week similarity with some putative GAG proteins in UniProt.

^*d *^This RT is from Mtr81 but can not be assigned to either superfamily.

To reveal the phylogenetic relationships within the superfamilies, we collected from literatures RT domains of 158 reference elements representing known Eukaryotic LTR lineages [21,29-31] and combined these data with our 74 families to construct trees [see Additional file 4]. We found that the Copia-like families belonged to 10 clades. MTC-1, a new lineage composed of Mtr5, Mtr49 and Mtr55, was recognized with middle support (Bootstrap value: 62%). The Gypsy-like families belonged to 4 well defined lineages (Figure 1).

### PBS pattern of LTR elements

We investigated TSD, PPT and PBS patterns of LTR elements. Although the TSDs and PPTs did not show significant sequence preference, we found clear tRNA usage bias through the PBS strings. The validation of a PBS sequence was to find the string which was located immediately downstream of the 5' LTR and a reverse complement to the 3' ends of a tRNA [32]. By the criterion of matching at least 14 bp, the PBSs were detected in 80 families, including all members in Copia and Gypsy superfamilies and 6 other families. We found that tRNAs corresponding to His, Phe, Tyr, Ser and Cys were never used as primer of reverse transcription and the majority of the rest 15 actually detected tRNA types occurred with low frequency. By contrast, the most-detected 4 types were used by nearly 3/4 families. Moreover, tRNA_*Met *_occurred in about 60% of the 80 families and was the most frequently used type in both superfamilies and TRIMs-LARDs. tRNA_*Arg *_was the second important primer in Gypsy superfamily and was only used by this group (Table 3 and [see Additional file 3]).

**Table 3:** tRNA usage of LTR families

tRNA type	Copia superfamily	Gypsy superfamily	Other	all
Met	34	6	2	42
Lys	4	1	2	7
Leu	5	0	1	6
Arg	0	4	0	4
Ala	1	2	0	3
Glu	1	2	0	3
Val	1	2	0	3
Asn	2	0	0	2
Ile	2	0	0	2
Tyr	2	0	0	2
Asp	0	1	1	2
Gln	1	0	0	1
Gly	1	0	0	1
Pro	1	0	0	1
Thr	1	0	0	1
SUM	56	18	6	80

### Copia superfamily

The present research discovered 64 novel families, including 38 Copia-like, 15 Gypsy-like and 11 other ones. We describe each group in the following three sections, with emphasis on some important families.

**Figure 2:**
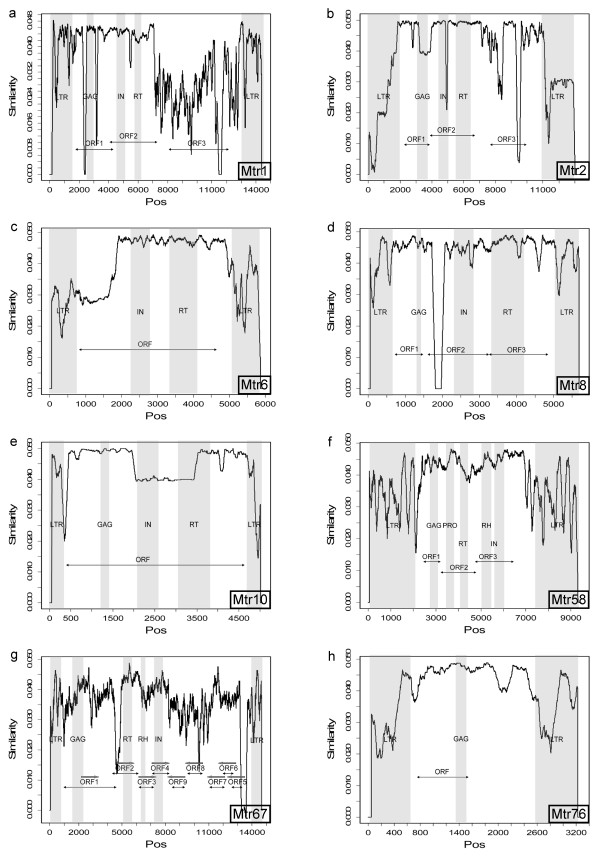
**Structure of LTR families**. Each sub-figure gives the structure of a family. the X-axis displays coordinates of nucleotides and the Y-axis displays average similarities among the full-length members of that family (calculated using the PLOTCON program in the EMBOSS package [46]). Grey stripes show the positions of the LTR and the domains detected. We display ORFs that are >500 bp in length. The arrow under a ORF label represents the length of that ORF. In Mtr67, the ORFs are found in both chains and their orientation is indicated by the arrows above the ORF labels. Sudden collapse of similarity (e.g. 1.8–2 Kb of Mtr8 and 2–3.5 Kb of Mtr10) is caused by the insertion or deletion events in one or two family members.

The total copy-number of the 56 Copia-like families reached 4816 and their sequences had a total length of 7164 Kb, about 3% of all genomic sequences investigated. Full-length elements varied in length from 3 to 12.7 Kb, with an average of 5.9 Kb. The longest family Mtr2/COPIA4, which had more than 130 copies, was one of the most abundant Copia-like families. We detected 3 long ORFs in its internal domain. From 5' to 3', the first two ORFs encoded canonical GAG-POL proteins. The third ORF, located less than 1 Kb downstream of the POL region, encoded a protein longer than 790AA. Although the homology search against Uniprot [33] did not return significant match to this ORF, it showed quite high conservation among the family members (Figure 2b). Moreover, we found that a 258 bp subregion in this ORF matched the putative ENV protein of the *Glycine max *putative endogenous retrovirus SIRE1-8 with low significance (similarity: 22%, e-value: 0.003). These results indicated that this ORF probably encoded a protein related to putative plant ENV.

Besides Mtr2, there were 6 Copia-like families longer than 10 Kb and all of them had the GAG, IN and RT domains. Mtr1 was the most abundant Copia-like family and the second largest in all the 85 families. It had 69 full-length members, more than 800 copies and its sequences reached more than 2.5 Mb in length, about 36% of the total length of the Copia-like copies. The percentage was 6.6 times as high as that of the second largest Copia-like family Mtr2, whose sequences were 392 Kb in length. The PBS of Mtr1 bounded tRNA_*Met *_and the length of its full-length members and LTRs were about 12 and 1.3 Kb, respectively. Outside *Mt*, the best match of its RT domain in Uniprot was from *V. vinifera *(Accession: A5BWH6). Similar to Mtr2, a third long ORF was also detected in its internal domain (Figure 2a). However, this ORF, matching a putative uncharacterized *Mt *protein (Q2HU06), was less conservative among the family members. The length of the LTRs of Copia-like families fell within the range of 126 bp to 2 Kb, with an average of 506 bp. LTRs could be roughly categorized into 4 zones: 100–500 bp, 600–850 bp, 1.2–1.45 Kb and >1.5 Kb. The number of families in these zones was 39, 8, 6 and 3, respectively [see Additional file 2]. We found that long elements tended to have long LTRs. In fact, almost all LTRs longer than 1 Kb were from elements longer than 10 Kb. Mtr3 and Mtr18 were exceptions. The length of the LTR of Mtr3 reached 1.28 Kb yet that of the full length was only 6 Kb. As one of the most abundant Copia-like retrotransposon, Mtr3 had 292 Kb sequences in the genome, It was a typical non-autonomous family since no >500 bp ORF could be detected in its internal domain and its RT degenerated to a fragment of 40AA.

There were 11 Copia-like families whose LTRs and full-length sequences were shorter than 200 bp and 5 Kb, respectively. 6 of them have been deposited in Repbase. Mtr30/MTCOPIA2 and Mtr32/COP3 were quite active in the genome and their copies corresponded to more than 200 and 109 Kb sequences, respectively. Despite short LTRs, we detected 5'-IN-RT-3' domains in all of them and GAG proteins in 7.

### Gypsy superfamily

The 18 Gypsy-like families corresponded to 11652 Kb sequences and constituted 5% of all genomic data. This group had a full length of 5.1 to 18.7 Kb, with an average of 9.8 Kb. Their LTRs were from 313 to 3.5 Kb in length, with an average of 1.5 Kb. Compared with Copia-like LTR retrotransposons, Gypsy-like elements were longer in general: the great majority of Gypsy-like families had a full length > 6 Kb and LTR >500 bp, while the full length and the LTR of most Copia-like families were < 6 Kb and 500bp, respectively [see Additional file 2]. The total copy-number in this superfamily was 7434, about 1.5 times more than Copia superfamily (4816). Despite fewer members, Gypsy superfamily contributed more to the *Mt *genome than Copia superfamily because of longer length and more active amplification in the past.

Mtr57 and Mtr59 were Ogre families [20,22] and Mtr57/Ogre1 was the largest in all the 85 families. Our survey detected its 114 full-length members and more than 1000 copies in total. The length of its sequences reached 4.2 Mb. Mtr70 was closely related to Mtr57 in the phylogenetic tree, and was the second longest in all the families (The longest one is Mtr57/Ogre1). These two families used tRNA_*Arg *_as primer and their internal domain encoded 5'-GAG-RT-RH-IN-3' proteins. We detected an ORF of 1527 bp located upstream of the GAG and an intron in the POL. Such phylogenetic and structural features well support that Mtr70 is a novel Ogre family. Mtr58 was the second largest family in the Gypsy group and the third largest in all the families. It had more than 600 copies in total and corresponded to 1.4 Mb sequences, about 1/3 of Mtr57/Ogre1. Its full length were about 8.8 Kb and the LTRs were 2.1 Kb in length. Its internal domain encoded 5'-GAG-PRO-RT-RH-IN-3' proteins (Figure 2f) and its PBS matched tRNA_*Met *_well. Phylogenetically, this family belonged to the Tekay clade.

Mtr67, Mtr60 and Mtr64 were the other 3 families longer than >10 Kb. Similar to Copia superfamily, it was found that, when the full-length of an element was >10 Kb, the LTR was correspondingly >1 Kb. The only exceptional family Mtr67 had a LTR of 720 bp. Besides normal 5'-GAG-RT-RH-IN-3' domains, this family had 5 additional >500 bp ORFs downstream of the POL. They were all located in the complementary chain and had no match in Uniport. However, these ORFs were quite conservative among the family members (Figure 2g). We estimated that these ORFs were derived from other sources and later captured by Mtr67. The short LTR reflected short original length of this family. Mtr60 belonged to the Athila clade (Figure 1). Its PBS bound tRNA_*Asp *_and its protein-coding domains organized as 5'-GAG-RT-IN-3'. Downstream of the POL, there were two >500 bp ORFs encoding uncharacterized proteins (best match in Uniprot: A2Q2P5 and A2Q2P6). Mtr64, the sister branch of Mtr60, also had an extra ORF downstream of the POL. Its best match in Uniprot was from *Garden asparagus *(Q2AA44) and it shared weak similarity with the first extra ORF in Mtr60 (Similarity: 24%, e-value: 2e-06). Known elements of the Athila clade were putative plant endogenous retroviruses, thus the possibility that the extra ORFs in Mtr60 and Mtr64 encoded the putative ENVs was strong, although they did not share significant similarity with the putative ENVs of known Athila elements.

Mtr65 and Mtr74 were from the CRM clade, while Mtr72/GYPSHAN and Mtr73 were from Renia. Families of these two clades were relatively inactive in *Mt*: each had a copy-number <50 and corresponded to <80 Kb sequences. Even so, they all showed multiple domains in their internal domain [see Additional file 3].

### TRIMs and LARDs

Because their internal domain lacked strong homology to any known TE proteins, the 11 families were required to have at least 5 strong hits in the genome. The total sequences of them were 3654 Kb in length, about 1.6% of all data. According to the suggestion of [3], the 5 families that had a length less than 4 Kb were classified as TRIMs and the other 6 as LARDs.

We detected ORFs longer than 700 bp in 4 families: Mtr80 had 2 such ORFs. One shared weak similarity with a GAG protein in rice (Q7XRT, 35%, 4 × 10^-92^) and the other with a putative transposon protein in *A. thaliana *(Q9XH30, 27%, 2 × 10^-8^). Although HMMER failed to detect (e-value: 10^-6^) RT and IN domains in 4 of the 5 full-length members, previous analyses suggested that Mtr80/MEGY belonged to a distinct clade called env-class [21]. Mtr78, Mtr82 and Mtr76 each had only one ORF >700 bp. The best Uniprot match of the ORF in Mtr78 was again Q9XH30 (24%, 2 × 10^-9^), while that of the ORF in Mtr82 was an uncharacterized protein from grape (AB5PC1, 46%, 3 × 10^-57^). The ORF in Mtr76 was highly similar to that in Mtr82 (80.1%) and a subregion in this ORF was reported as a GAG by HMMER (Figure 2h).

Mtr81 is the longest family in the 11. Although its internal domain was long, most members in this family rarely contained long ORFs. Searching the internal domain against Uniprot with BLASTX retrieved a 438 bp region homologous to some RT proteins and the best well-studied match was from the maize (*Zea mays*) Opie element (Q8H7T1, 60%, 1 × 10^-48^). The high similarity with Opie strongly supported that this ORF did encode a RT domain. However, it could not be detected by HMMER and this indicated that this RT might be not built in current RT profiles. Phylogenetic analysis further revealed that it belonged to neither Copia nor Gypsy superfamily but was close to the outgroup [see Additional file 5]. Further investigation is needed to fully resolve its position.

The last family that shared homology with known TE proteins was Mtr85. It had a ~321 bp ORF encoding a fragment of RH (A4PUT7, similarity: 91%, e-value: 1 × 10^-49^). Each of the other 5 families had more than 10 strong hits and quite large copy-number in the genome. Mtr75 was the shortest family, of which the full-length members and the LTRs were only 364 and 130 bp in length, respectively. Instead of typical 5'-TG-3', 5' ends of its LTRs were 5'-TA-3'. Despite highly degenerated internal domain, this family had 9 full-length members and 74 strong hits. Mtr77, Mtr83 and Mtr84 were LARDs. Similar to Mtr75, Mtr77 was an abundant non-autonomous family with highly degenerated internal domain (about 200 bp).

### Structure of LTR retrotransposons in *Mt*

We studied the structure of the LTR families and Figure 2 displays the structures of 8 highly abundant ones, five of which have been described above. The structure of Ogre elements is not shown because it has been reported previously [22]. Here we just point out that the LTR regions of most families tend to be less conservative among the family members in comparison with TE proteins, as well as many long ORFs. This result well supports that LTRs are the most rapidly evolving regions in LTR retrotransposons [3,34].

### Insertion-deletion of LTR retrotransposons in *Mt*

Mtr1, Mtr2, Mtr6, Mtr10, Mtr57-59 and Mtr76 each had more than 10 full-length members. The total number of these full-length copies was 296, making up 56.3% of all the full-length elements identified. Their total copies constituted 45.3% of the LTR retrotransposon sequences.

Paleontology analysis on the 296 elements revealed that they were quite young: all were inserted within the last 2 MY and 90% within 0.4 MY. Compared with others, the active period of Mtr6 (10 full-length copies) and Mtr76 (17 full-length copies) was relatively long [see Additional file 6]. Recent researches have argued that truncated LTR elements were mainly caused by unequal homologous or illegitimate recombination within genome and the result of recombination was the deletion of genomic sequences [27,32,35,36]. We estimated the deletion of LTR copies in *Mt *genome from two aspects: 1) the deletion of full-length structure and 2) the number of DNA loss. The deletion of a full-length structure means that mutation and recombination remove so many structural characters of a full-length element that it can not be recognized any more. Assuming that repetitive sequences are removed at a constant rate, the survival time of full-length structure obeys an exponential distribution and therefore the half-life is an index to estimate the speed of removal. With this method, [32] estimated that the half-life of the full-length elements in rice was about 0.79 MY.

**Figure 3:**
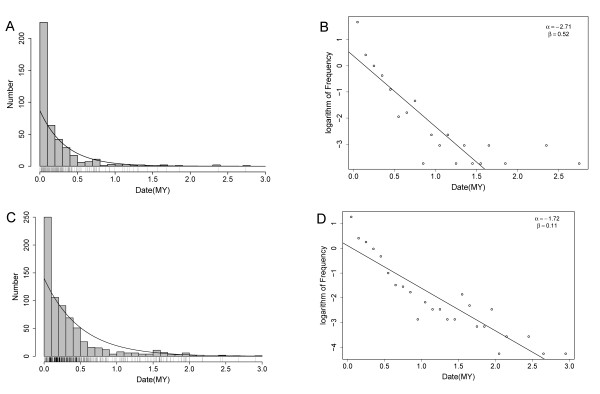
**Half-life of full-length LTR retrotransposons in *Mt *and rice**. 526 *Mt *and 705 rice full-length elements are analyzed. Each bin represents 0.1 MY. Vertical bars under the histogram represent insertion events. a) The distribution of the insertion date of *Mt *elements. Fitting of this distribution to a exponential curve indicates that the insertions in the recent 0.1 MY have been significantly active. b) Fitting the dates to the exponential curve. The logarithm of the dates fits the straight line *y *= 0.52 - 2.71*x *well. Therefore the rate of the exponential curve is *α *= -2.71, which corresponds to a half-life of 0.26 MY. c) and d) display the fitting in rice, which gives a half-life of 0.4 MY.

We calculated the insertion date of all the 526 full-length elements and found that 90% of them inserted within the last 0.52 MY (Figure 3a). Fitting of the distribution to the exponential function obtained *α *= -2.71, which corresponded to a half-life *τ *= 0.26 MY (Figure 3b). The bootstrap revealed that the half-life varied between 0.24 to 0.3 MY. To compare the speed of removal in legume and grass, we calculated the half-life for 705 full-length elements in the two sequenced rice genomes (*Oryza sativa indica ssp*. and *japonica ssp*.). Elements in rice and *Mt *were predicted under the same parameters (Hao Wang, unpublished data). As can be seen from Figure 3c and 3d, our data supported that the half-life of full-length structure in rice was ~0.4 MY, a lesser value than the estimation of [32] but still greater than that in *Mt*. Furthermore, statistical testing revealed that the insertion dates in the two species were from different distributions (Kolmogorov-Smirnov test, P-Value: 3.4 × 10^-14^). If the mean substitution rates of LTR elements in *Mt *and grass are approximately the same [37], the above results support that the full-length structures have been deleted more rapid in *Mt *than in rice. If the deletions have been occurred randomly in genome, the results further indicate that the removal of LTR elements in *Mt *has been more rapid than in rice.

The total number of the strong hits was only 6% of all detected copies, but their size reached 42%. This indicated that LTR elements in *Mt *were highly fragmented and these truncated copies, great in number, might be generated by the removal of genomic DNA. If the truncated LTR copies were real vestiges of paralogous copies of families and if they had similar lengthes to the representative copies at the time of insertion, the difference of the length of truncated and representative copies provided the amount of deleted DNA since their insertion [36]. The estimation of the upper and lower limits of DNA loss could be as follows: we used the copies of Rset (see Methods) to estimate the lower limit. The data revealed that 5.5 Mb sequences have been deleted. Since Rset only consisted of not-so-severely truncated copies, it caused an underestimate of DNA loss. In contrast, we used all the truncated copies to estimate the upper limit and this gave more than 46 Mb of DNA has been removed. Since only 40% of the genome was analyzed here, we estimated that more than 10 Mb of LTR retrotransposon sequences have been deleted from the *Mt *genome.

## Conclusion

We have systematically identified and described LTR retrotransposons in nearly half of the *Medicago truncatula *genome, investigated their classification, structure, evolutionary dynamics and impact on the evolution of the host genome. The present work has provided a LTR retrotransposon landscape for this model legume. The sequencing of other species such as *Lotus japonicus *and *Glycine max *will provide great opportunity to study comparatively the evolutionary dynamics of LTR families in two or more legume organisms and further explore the interactions between these elements and their host genomes.

## Methods

### Genomic sequences, LTR element databases and tRNA database

The *Mt *genomic data were composed of 1826 BACs, about 233 Mb in length. The data were downloaded from *Medicago truncatula *Sequencing Resources website (Version 1.0. Released on July, 2006) [38].

A database of known legume LTR retrotransposons was constructed by extracting legume elements from literatures [21,29-31], Repbase [23] and TIGR Plant Repeat Databases [24]. This database was used to discriminate previously reported families from novel ones discovered in this research. A *Mt *tRNA database was also built by scanning the genome with tRNAscan-SE [39]. It was used to detect PBS of LTR elements by LTR_FINDER, our newly developed *ab initio *tool for the prediction of full-length LTR retrotransposons [25].

### Mining LTR retrotransposons in *Medicago truncatula *genome

We first identified candidates of full-length element with LTR_FINDER, then annotated other copies related to them in the genome by homology search and the elimination of pseudo-copies. At last, only the candidates that had multiple copies were kept as LTR retrotransposons for further analysis.

The initial LTR_FINDER scan retrieved more than 600 candidates. They were then subjected to the following steps to validate copies.

**Figure 4:**
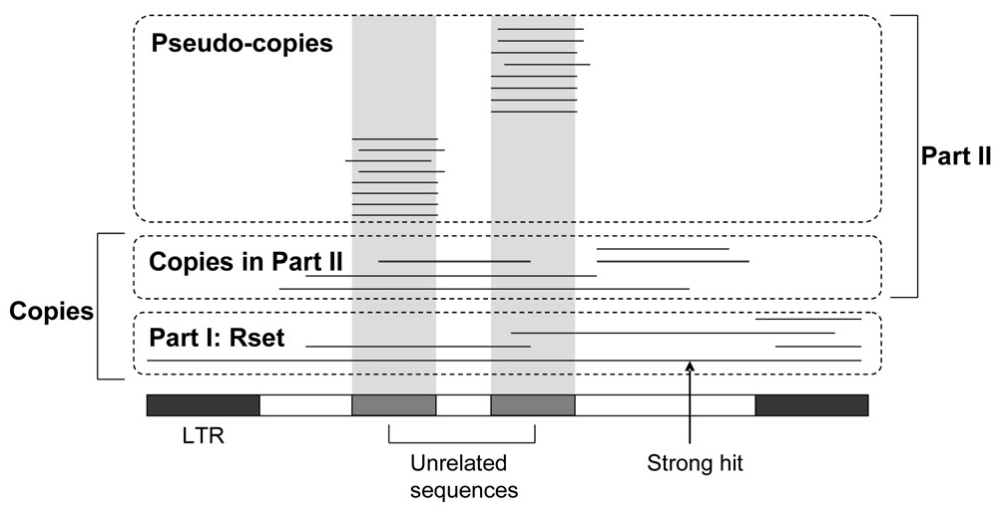
**Homologous matches of an candidate**. The lines represent matches generated by whole-genome homology search of a reference candidate. Some matches are made of several pieces (segments on the same horizontal line). All the matches are categorized into Part I and Part II. Part I (Rset) consists of the matches that cover both the LTR region and the internal domain. They are reliable copies of the reference candidate. Part II is further classified into pseudo-copies and "copies in part II". Pseudo-copies are the matches that correspond to unrelated sequences. Unrelated sequences (dark grey regions) are the subregions that have significantly high matches (grey stripes) or that match some LTR elements well (not showing here). At last, "copies in part II" and Rset are combined to obtain the total copies of the candidate.

1. Selected reliable LTR copies of candidates (Rset). The *Mt *genome was searched against each LTR candidate and all the matches longer than 100 bp were taken as the basic set of copies related to that candidate. This basic set were partitioned into two sets: Part I and Part II. Part I (called Rset) consisted of matches that covered both the LTR region and internal domain to a certain length (Figure 4), which, obviously, was a subset of all copies because it excluded severely truncated ones. Part II consisted of other matches. Since this set might contain pseudo-copies generated by unrelated sequences, it is processed by the following two steps to eliminate pseudo-copies.

2. Detected unrelated sequences derived from other LTR elements and eliminated their pseudo-copies. The internal domain of each candidate was searched against all the candidates to find whether it contained other LTR retrotransposons. The subregions derived from other candidates were recorded as unrelated sequences. Accordingly, the matches generated by such subregions were eliminated from Part II.

3. Detected unrelated sequences derived from other TEs and eliminated their pseudo-copies. When an unrelated sequence was derived from an abundant TE, it would have many matches in the genome. Therefore, if the subregions of the internal domain had significantly high number of matches, the possibility that they were unrelated sequences was high (Figure 4). We used a sliding window to find such subregions:

(a) A window of size w (say 100 bp) moved from 5' to 3' along the internal domain and stopped at the first position that at least k (say 20) hits were found in it. Here a hit was a member of Part II that covered at least 80% of the window.

(b) The window extended 1 bp and was checked if it still had k (or more) hits. The extension continued until less than k hits were in the window, then the current window was marked and this completed a search cycle. the next cycle started from the next position to the 3' end of the window. The search continued until the window reached the 3' end of the internal domain.

(c) All of the marked windows were checked to filter those that obviously overlapped with TE proteins. Subsequently, the remaining windows were connected if the distances between them were less than a threshold (say 10 bp). At last, the regions covered by such windows were taken as unrelated sequences and their corresponding matches were eliminated from Part II (Figure 4).

Although this simple greedy algorithm might fail to deal with a few complicated situations, it identified most of the pseudo-copies efficiently.

4. Obtained copies of candidates. Rset and the remaining copies in Part II were combined to obtain the copies of the candidates. If one locus in the genome matched several candidates, it was assigned to the best matched one. At last, a full-length candidate was taken as a LTR element if it had several hits covering at least 80% of it. Such hits are called strong hits or strong-hit copies. After the above validation, a total of 526 full-length elements and their 16565 related copies were selected. To be reliable, the above validation excluded the matches that were shorter than 100 bp. This might skip some severely truncated copies and thus brought some underestimation of DNA loss and the contribution of LTR retrotransposons to the genome.

Subsequently, full-length elements were categorized into families by their sequence similarity [3], and the copies of each family were obtained by combining all the copies of its full-length members. The annotation process identified unrelated TE sequences in elements and discarded pseudo-copies, thus estimated the contribution of LTR elements to the genome more accurately.

### TE domain identification

LTR_FINDER tried to detect ORFs of RT, IN and RH in the full-length elements by calling PS_SCAN [25,40]. Besides this, we scanned them (e-value: 10^-6^) with the hmmsearch program in the HMMER package [41] to locate positions of important domains. The profiles were downloaded directly from Pfam (V22.0) [42]. According to the suggestion of [26], TE domains were represented by the following profiles: RT by PF00078, PF07727 and PF05380; IN by PF00665, PF00552 and PF02022; PRO by PF00026 and PF00077; RH by PF00075; GAG by PF03732 and PF00098; and ENV by PF03078. We note that the scan process may skip some domains if they are highly divergent among different retrotransposon families or not built in Pfam profiles.

### Phylogenetic and statistical analysis

The phylogenetic tree and multiple alignments were constructed by CLUSTALW [43]. The tree was edited with MEGA4 [44]. Statistical analyses were performed by R [45]. Following the suggestion of [37], 1.3 × 10^-8^/*site*/*yr *was used as the average substitution rate of *Mt *LTR elements to obtain the insertion date of elements.

## Authors' contributions

HW designed and carried out the LTR retrotransposon studies, participated in the design of LTR retrotransposon mining program and drafted the manuscript. J-SL participated in the design of the program of LTR copy validation. All authors read and approved the final manuscript.

## Supplementary Material

Additional file 1 **Sequences of 526 full-length LTR elements identified in this study**. this file contains the sequences of all full-length LTR elements identified in this study. Click here for file

Additional file 2 **Distribution of the length of Copia and Gypsy superfamilies**. this file contains two figures showing the distributions of the full-length and LTR length of *Mt *elements. Click here for file

Additional file 3 **Domain structure and PBS usage of LTR families**. this file provides the information to relate the LTR families with their protein domain structures and reverse transcription primer tRNAs. Click here for file

Additional file 4 **Phylogeny of Copia- and Gypsy-like LTR families**. this file contains the phylogenetic analysis of Copia and Gypsy superfamilies. Click here for file

Additional file 5 **Phylogenetic position of Mtr81**. the RT sequence of Mtr81 do not belongs to Copia or Gypsy superfamily. It is placed as a third branch the phylogenetic tree. Click here for file

Additional file 6 **Insertion dates of 8 abundant LTR families**. this file contains a figure showing the distribution of the insertion time of 8 abundant LTR families. Click here for file

## References

[B1] Kim JM, Vanguri S, Boeke JD, Gabriel A, Voytas DF (1998). Transposable elements and genome organization: a comprehensive survey of retrotransposons revealed by the complete Saccharomyces cerevisiae genome sequence. Genome Res.

[B2] Ganko EW, Fielman KT, McDonald JF (2001). Evolutionary history of Cer elements and their impact on the C. elegans genome. Genome Res.

[B3] Wicker T, Sabot F, Hua-Van A, Bennetzen JL, Capy P, Chalhoub B, Flavell A, Leroy P, Morgante M, Panaud O, Paux E, SanMiguel P, Schulman AH (2007). A unified classification system for eukaryotic transposable elements. Nat Rev Genet.

[B4] McCarthy EM, McDonald JF (2003). LTR STRUC: a novel search and identification program for LTR retrotransposons. Bioinformatics.

[B5] Flavell RB (1986). Repetitive DNA and chromosome evolution in plants. Philos Trans R Soc Lond B Biol Sci.

[B6] Meyers BC, Tingey SV, Morgante M (2001). Abundance, distribution, and transcriptional activity of repetitive elements in the maize genome. Genome Res.

[B7] McCarthy EM, Liu J, Lizhi G, McDonald JF (2002). Long terminal repeat retrotransposons of Oryza sativa. Genome Biol.

[B8] SanMiguel P, Tikhonov A, Jin YK, Motchoulskaia N, Zakharov D, Melake-Berhan A, Springer PS, Edwards KJ, Lee M, Avramova Z, Bennetzen JL (1996). Nested retrotransposons in the intergenic regions of the maize genome. Science.

[B9] Wicker T, Stein N, Albar L, Feuillet C, Schlagenhauf E, Keller B (2001). Analysis of a contiguous 211 kb sequence in diploid wheat (Triticum monococcum L.) reveals multiple mechanisms of genome evolution. Plant J.

[B10] Hawkins JS, Kim H, Nason JD, Wing RA, Wendel JF (2006). Differential lineage-specific amplification of transposable elements is responsible for genome size variation in Gossypium. Genome Res.

[B11] Piegu B, Guyot R, Picault N, Roulin A, Saniyal A, Kim H, Collura K, Brar DS, Jackson S, Wing RA, Panaud O (2006). Doubling genome size without polyploidization: dynamics of retrotransposition-driven genomic expansions in Oryza australiensis, a wild relative of rice. Genome Res.

[B12] Kapitonov VV, Jurka J (1999). The long terminal repeat of an endogenous retrovirus induces alternative splicing and encodes an additional carboxy-terminal sequence in the human leptin receptor. J Mol Evol.

[B13] Ganko EW, Bhattacharjee V, Schliekelman P, McDonald JF (2003). Evidence for the contribution of LTR retrotransposons to C. elegans gene evolution. Mol Biol Evol.

[B14] Du C, Swigonova Z, Messing J (2006). Retrotranspositions in orthologous regions of closely related grass species. BMC Evol Biol.

[B15] Wang W, Zheng H, Fan C, Li J, Shi J, Cai Z, Zhang G, Liu D, Zhang J, Vang S, Lu Z, Wong GKS, Long M, Wang J (2006). High rate of chimeric gene origination by retroposition in plant genomes. Plant Cell.

[B16] Graham P, Vance C (2003). Legumes: importance and constraints to greater use. Plant Physiol.

[B17] Cannon SB, Sterck L, Rombauts S, Sato S, Cheung F, Gouzy J, Wang X, Mudge J, Vasdewani J, Schiex T, Scheix T, Spannagl M, Monaghan E, Nicholson C, Humphray SJ, Schoof H, Mayer KFX, Rogers J, Quétier F, Oldroyd GE, Debellé F, Cook DR, Retzel EF, Roe BA, Town CD, Tabata S, de Peer YV, Young ND (2006). Legume genome evolution viewed through the Medicago truncatula and Lotus japonicus genomes. Proc Natl Acad Sci USA.

[B18] Vitte C, Panaud O (2005). LTR retrotransposons and flowering plant genome size: emergence of the increase/decrease model. Cytogenet Genome Res.

[B19] Grzebelus D, Lasota S, Gambin T, Kucherov G, Gambin A (2007). Diversity and structure of PIF/Harbinger-like elements in the genome of Medicago truncatula. BMC Genomics.

[B20] Neumann P, Požárková D, Macas J (2003). Highly abundant pea LTR retrotransposon Ogre is constitutively transcribed and partially spliced. Plant Molecular Biology.

[B21] Neumann P, Pozárková D, Koblízková A, Macas J (2005). PIGY, a new plant envelope-class LTR retrotransposon. Mol Genet Genomics.

[B22] Macas J, Neumann P (2007). Ogre elements-a distinct group of plant Ty3/gypsy-like retrotransposons. Gene.

[B23] Genetic Information Research Institute. http://www.girinst.org/.

[B24] TIGR Plant Repeat Databases. http://www.tigr.org/tdb/e2k1/plant.repeats/.

[B25] Xu Z, Wang H (2007). LTR_FINDER: an efficient tool for the prediction of full-length LTR retrotransposons. Nucleic Acids Res.

[B26] Rho M, Choi JH, Kim S, Lynch M, Tang H (2007). De novo identification of LTR retrotransposons in eukaryotic genomes. BMC Genomics.

[B27] Ma J, Bennetzen JL (2004). Rapid recent growth and divergence of rice nuclear genomes. Proc Natl Acad Sci USA.

[B28] Kapitonov VV, Jurka J (1999). Molecular paleontology of transposable elements from Arabidopsis thaliana. Genetica.

[B29] Kordiš D (2005). A genomic perspective on the chromodomain-containing retrotransposons: Chromoviruses. Gene.

[B30] Macas J, Neumann P, Navrátilová A (2007). Repetitive DNA in the pea (Pisum sativum L.) genome: comprehensive characterization using 454 sequencing and comparison to soybean and Medicago truncatula. BMC Genomics.

[B31] Lloréns C, Futami R, Bezemer D, Moya A (2008). The Gypsy Database (GyDB) of mobile genetic elements. Nucleic Acids Res.

[B32] Wicker T, Keller B (2007). Genome-wide comparative analysis of copia retrotransposons in Triticeae, rice, and Arabidopsis reveals conserved ancient evolutionary lineages and distinct dynamics of individual copia families. Genome Res.

[B33] UniProt (Universal Protein Resource). http://www.expasy.org/.

[B34] McCarthy EM, McDonald JF (2004). Long terminal repeat retrotransposons of Mus musculus. Genome Biol.

[B35] Devos KM, Brown JK, Bennetzen JL (2002). Genome size reduction through illegitimate recombination counteracts genome expansion in Arabidopsis. Genome Res.

[B36] Vitte C, Panaud O, Quesneville H (2007). LTR retrotransposons in rice (Oryza sativa, L.): recent burst amplifications followed by rapid DNA loss. BMC Genomics.

[B37] Vitte C, Bennetzen JL (2006). Analysis of retrotransposon structural diversity uncovers properties and propensities in angiosperm genome evolution. Proc Natl Acad Sci USA.

[B38] *Medicago truncatula *Sequencing Resources website. http://www.medicago.org/.

[B39] Lowe TM, Eddy SR (1997). tRNAscan-SE: a program for improved detection of transfer RNA genes in genomic sequence. Nucleic Acids Res.

[B40] Gattiker A, Gasteiger E, Bairoch A (2002). ScanProsite: a reference implementation of a PROSITE scanning tool. Appl Bioinformatics.

[B41] Durbin R, Eddy S, Krogh A, Mitchison G (1998). Biological sequence analysis: probabilistic models of proteins and nucleic acids.

[B42] Finn RD, Mistry J, Schuster-Böckler B, Griffiths-Jones S, Hollich V, Lassmann T, Moxon S, Marshall M, Khanna A, Durbin R, Eddy SR, Sonnhammer ELL, Bateman A (2006). Pfam: clans, web tools and services. Nucleic Acids Res.

[B43] Thompson JD, Higgins DG, Gibson TJ (1994). CLUSTAL W: improving the sensitivity of progressive multiple sequence alignment through sequence weighting, position-specific gap penalties and weight matrix choice. Nucleic Acids Res.

[B44] Tamura K, Dudley J, Nei M, Kumar S (2007). MEGA4: Molecular Evolutionary Genetics Analysis (MEGA) software version 4.0. Mol Biol Evol.

[B45] The R Project for Statistical Computing. http://www.r-project.org/.

[B46] Rice P, Longden I, Bleasby A (2000). EMBOSS: the European Molecular Biology Open Software Suite. Trends Genet.

