# Supplementary material for: LTR retrotransposon landscape in Medicago truncatula: more rapid removal than in rice
Source: BMC Genomics. 2008 Aug 10;9:382. doi: 10.1186/1471-2164-9-382 (PMC2533021; doi:10.1186/1471-2164-9-382)
Supplement: Additional file 2 — Distribution of the length of Copia and Gypsy superfamilies. this file contains two figures showing the distributions of the full-length and LTR length of Mt elements. [file 1471-2164-9-382-S2.pdf]

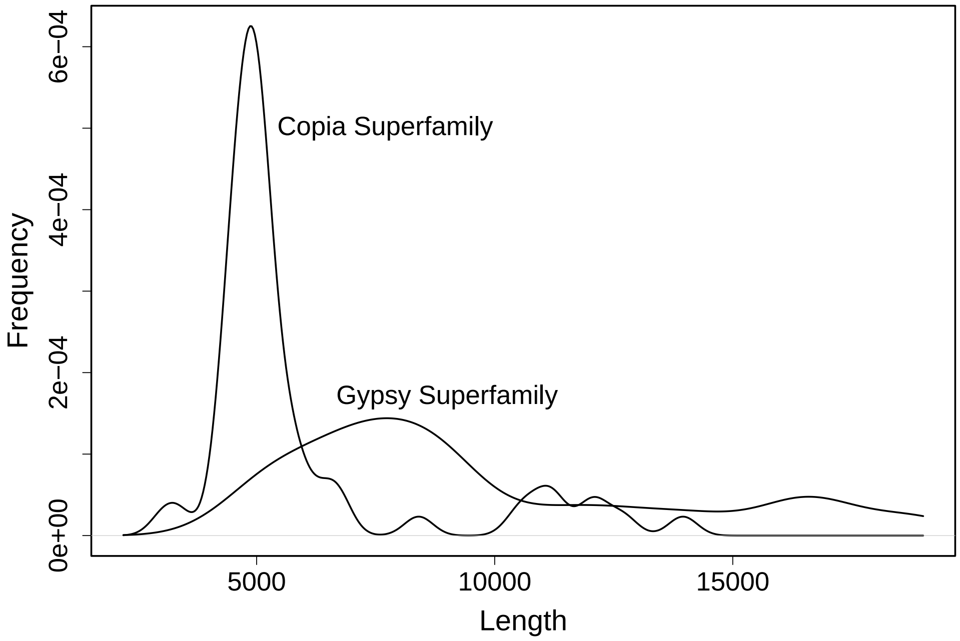

(a) The distribution of full length.

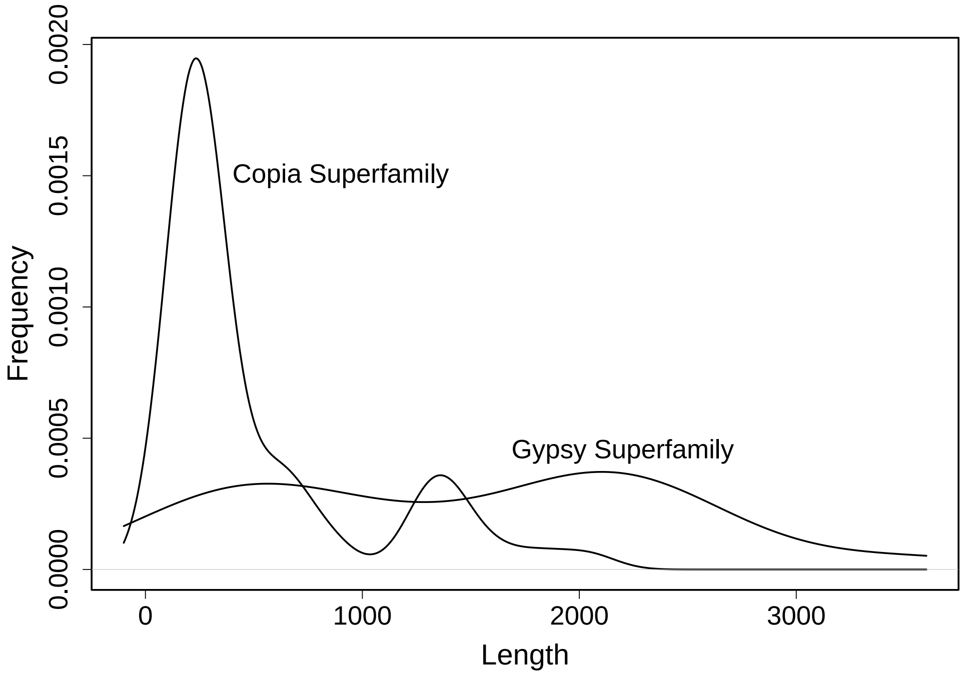

(b) The distribution of the length of LTR.

Figure S2.1: The distribution of the length of full-length element and LTR in Copia and Gypsy superfamilies.
