# Supplementary material for: LTR retrotransposon landscape in Medicago truncatula: more rapid removal than in rice
Source: BMC Genomics. 2008 Aug 10;9:382. doi: 10.1186/1471-2164-9-382 (PMC2533021; doi:10.1186/1471-2164-9-382)
Supplement: Additional file 3 — Domain structure and PBS usage of LTR families. this file provides the information to relate the LTR families with their protein domain structures and reverse transcription primer tRNAs. [file 1471-2164-9-382-S3.pdf]

Table S3.1: Domain structure and PBS usage of LTR families.

Family	PBS	Domain
Mtr1	GAG-IN-RT	Met
Mtr2	GAG-IN-RT	Met
Mtr3	RT	Met
Mtr4	GAG-IN-RT	Ile
Mtr5	RT	Met
Mtr6	GAG-IN-RT	Lys
Mtr7	GAG-IN-RT	Asn
Mtr8	GAG-IN-RT	Lys
Mtr9	GAG-IN-RT	Met
Mtr10	GAG-IN-RT	Met
Mtr11	GAG-IN-RT	Pro
Mtr12	GAG-IN-RT	Met
Mtr13	GAG-IN-RT	Met
Mtr14	GAG-IN-RT	Met
Mtr15	GAG-IN-RT	Met
Mtr16	GAG-IN-RT	Met
Mtr17	IN-RT	Met
Mtr18	GAG-IN-RT	Lys
Mtr19	GAG-IN-RT	Met
Mtr20	IN-RT	Met
Mtr21	GAG-IN-RT	Met
Mtr22	GAG-IN-RT	Met
Mtr23	IN-RT	Leu
Mtr24	GAG-IN-RT	Met
Mtr25	GAG-IN-RT	Met
Mtr26	GAG-IN-RT	Ala
Mtr27	GAG-IN-RT	Met
Mtr28	IN-RT	Tyr
Mtr29	IN-RT	Asn
Mtr30	GAG-IN-RT	Met
Mtr31	IN-RT	Met
Mtr32	IN-RT	Leu
Mtr33	GAG-IN-RT	Met
Mtr34	IN-RT	Met
Mtr35	GAG-IN-RT	Met
Mtr36	GAG-IN-RT	Met
Mtr37	IN-RT	Leu
Mtr38	IN-RT	Met
Mtr39	IN-RT	Glu
Mtr40	GAG-IN-RT	Gln
Mtr41	GAG-IN-RT	Tyr
Mtr42	GAG-IN-RT	Met
Mtr43	GAG-IN-RT	Met
Mtr44	IN-RT	Met
Mtr45	GAG-IN-RT	Met
Mtr46	IN-RT	Leu

(continued)

Family	PBS	Domain
Mtr47	GAG-IN-RT	Ile
Mtr48	GAG-IN-RT	Met
Mtr49	IN-RT	Gly
Mtr50	GAG-IN-RT	Leu
Mtr51	GAG-IN-RT	Lys
Mtr52	IN-RT	Met
Mtr53	IN-RT	Met
Mtr54	IN-RT	Val
Mtr55	IN-RT	Thr
Mtr56	IN-RT	Met
Mtr57	GAG-RT-RNaseH-IN	Arg
Mtr58	GAG-Protease-RT-IN	Met
Mtr59	GAG-RT-RNaseH-IN	Arg
Mtr60	GAG-RT-IN	Asp
Mtr61	GAG-Protease-RT-IN	Ala
Mtr62	GAG-Protease-RT	Met
Mtr63	RT-IN	Met
Mtr64	GAG-RT-IN	Glu
Mtr65	GAG-RT-IN	Val
Mtr66	RT-IN	Met
Mtr67	GAG-RT-RNaseH-IN	Arg
Mtr68	GAG-RT-RNaseH-IN	Lys
Mtr69	RT-IN	Met
Mtr70	GAG-RT-RNaseH-IN	Arg
Mtr71	GAG-Protease-RT-IN	Met
Mtr72	GAG-RT-IN	Val
Mtr73	GAG-RT-IN	Glu
Mtr74	GAG-RT-IN	Ala
Mtr75	ND	ND
Mtr76	GAG	Lys
Mtr77	ND	ND
Mtr78	ND	Met
Mtr79	ND	ND
Mtr80	GAG	Asp
Mtr81	RT	Met
Mtr82	GAG	Lys
Mtr83	ND	ND
Mtr84	ND	ND
Mtr85	ND	Leu
