# Supplementary material for: LTR retrotransposon landscape in Medicago truncatula: more rapid removal than in rice
Source: BMC Genomics. 2008 Aug 10;9:382. doi: 10.1186/1471-2164-9-382 (PMC2533021; doi:10.1186/1471-2164-9-382)
Supplement: Additional file 4 — Phylogeny of Copia- and Gypsy-like LTR families. this file contains the phylogenetic analysis of Copia and Gypsy superfamilies. [file 1471-2164-9-382-S4.pdf]

Phylogeny of Copia- and Gypsy-like LTR families

Hao Wang and Jin-Song Liu

1 Phylogeny of Gypsy Superfamily

We have collected 52 reference elements (Table S4.1) representing known Gypsy clades in Eukaryotic organisms (mainly in plants and fungi) and combined them with the 18 Gypsy-like *Mt* LTR families detected in this study. The NJ tree of the 70 families is shown in Figure S4.1 (BEL-1-I_NVp is the outgroup). The *Mt* Gypsy-like families fall into 4 plant LTR clades: Tekay, CRM, Reina and Athila/Tat (Kordiš 2005, Macas and Neumann 2007, Lloréns et al. 2008). This 70-family tree shows consistency with that constructed by our 18 Gypsy-like families (Figure 1).

Table S4.1: List of reference elements in Gypsy superfamily.

Clades	Name	Accession	Species
Tekay	Del1-46	X13886	L._henryi
	Legolas	AC006570	A._thaliana
	RIRE3	AB014738	O._sativa
	Ljchromovir-4	AP004471	L._japonicus
	Mtchromovir-2	AC144805	M._truncatula
	Leviathan-1	AY144442	S._bicolor
	Tekay	AF448416	Z._mays
	Peabody	AF083074	P._sativum
	Retrosat-2	AF111709	O._sativa
Reina	Gimli	AL049655	A._thaliana
	IFG7	AJ004945	P._radiata
	Mtchromovir-1	AC124965	M._truncatula
	Ljchromovir-5	AP004517	L._japonicus
	Ljchromovir-12	AP004473	L._japonicus
	Reina	U69258	Z._mays
CRM	RIRE7	AB033235	O._sativa
	LjCRM-1	AP004525	L._japonicus
	Beetle-1	AJ539424	B._vulgaris
Galadriel	Galadriel	AF119040	L._esculentum
	Pop1-2C5	AC149542	P._balsamifera
Mgrl3	Mgrl-3	AF314096	M._grisea
	Real	AB025309	A._alternata
Coccy	Coccy-1	AAEC01000102	C._immitis
	Coccy-2	AAEC01000098	C._immitis
Pyret	Pyret	AB062507	M._grisea
	Skippy	L34658	F._oxysporum

(continued)

Clades	Name	Accession	Species
	Cft1	Z11866	C._fulvum
Tf1	Tf2	L10324	S._pombe
Ty3	SpTy3-1	AABY01000202	S._paradoxus
	SkTy3-1	AACI01000169	S._kudriavzevii
	ScTy3-1	AACF01000040	S._castellii
MarY1	MarY1	AB028236	T._matsutake
	Ccchromovir-4	AACS01000165	C._cinerea
Tcn1	Ccchromovir-1	AACS01000152	C._cinerea
	Ccchromovir-2	AACS01000277	C._cinerea
Tcn2	Ccchromovir-5	AACS01000005	C._cinerea
Smut	Uhchromovir-1	AC114899	U._hordei
Sushi	Drsushi-33	AL596141	D._rerio
	Gasushi-1	AC145765	G._aculeatus
Skipper	Skipper	AF049230	D._discoideum
Oovir	Pichromovir-1	AC146942	P._infestans
	Pichromovir-2	AC147180	P._infestans
	Pichromovir-6	AC146942	P._infestans
Athila	Athila4-1	AC007209	A._thaliana
	Bagy-2	AF254799	H._vulgar
	Cyclops-2	AJ000640	P._stivum
	Diaspora	AF095730	G._max
Tat	ACinful-1	F049110	Z._mays
	Grande1-4	X97604	Z._diploperennis
	Ogre	AY299398	P._sativum
	RetroSor1	AF098806	S._bicolor
	RIRE2	AB030283	O._sativa

2 Phylogeny of Copia Superfamily

The phylogeny of Copia superfamily is less sophisticated. Macas et al. (2007) have provided an update analysis, which recognized 8 Copia clades (Macas et al. 2007). We have combined our 56 *Mt* families with 106 Repbase elements (Table S4.2) representing these clades and constructed the NJ tree. As can be seen from Figure S4.2, the 56 Copia-like families distribute in all the 8 clades. We recognize a new clade, named MTC-1, with middle supports (bootstrap value: 62%). We find that the poorly supported lineage Clade 7 (bootstrap value 47% in (Macas et al. 2007)) is paraphyletic. However, its two daughter clades were considerably supported. We name them as Clade7.1 and Clade7.2, respectively. Although MTC-1 and Clade7.1 show to be sister clades in the tree, the grouping of them is rather poorly supported (bootstrap value: 27%). Therefore we take them as distinct clades.

This 162-family phylogeny shows high consistency with Figure 1. The only difference is the placement of the (Mtr3, Mtr39) lineage. This lineage is poorly supported (47%) in Figure S4.2. However, these two families do not form a clade in figure 1. This disagreement suggests further research is needed to resolve their positions.

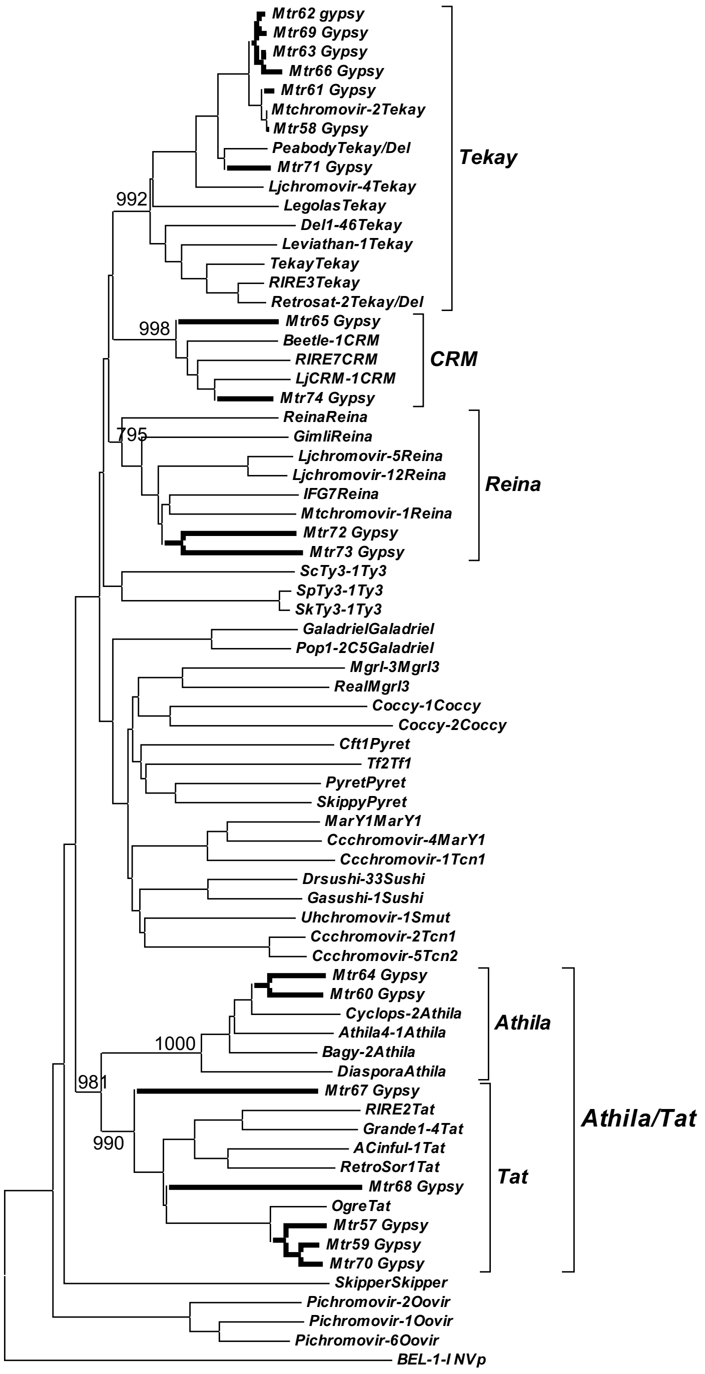

Figure S4.1: Tree of Gypsy superfamily. 1000 bootstraps have been performed. Only the stability of the 4 clades are shown. The labels of reference sequences are formatted as the element name directly followed by the clade id.

Table S4.2: List of reference elements in Copia superfamily.

Clades	Accession	Species
Outgroup	Copia3-I_XT	X._tropicalis
No marked	COP2_I_MT	M._truncatula
clade1	PGCOPIAX1	P._glauca
	ATCOPIA37_I	A._thaliana
	COP7_I_MT	M._truncatula
	ATCOPIA49_I	A._thaliana
	ATCOPIA69_I	A._thaliana
	ATCOPIA33_I	A._thaliana
	ATCOPIA76_I	A._thaliana
	ATCOPIA32_I	A._thaliana
	TA1-2_I	A._thaliana
	ATCOPIA10_I	A._thaliana
	COP18_I_MT	M._truncatula
	SHACOP17_I_MT	M._truncatula
	COPIA3-I_OS	O._sativa
	COPI1_I	O._sativa
	SHACOP_I_MT	M._truncatula
	SHACOP15_I_MT	M._truncatula
	SHACOP16_I_MT	M._truncatula
	TLC1_I	L._chilense
clade2	ZMCOPIA2_I	Z._mays
	TAR1_TA	T._aestivum
	RIRE5-I_OS	O._sativa
	COP16_I_MT	M._truncatula
	TONT2-I_PV	P._vulgaris
	COP8_I_MT	M._truncatula
	TONT1_LE_I	L._esculentum
	ATCOPIA95_I	A._thaliana
clade3	RN107_I	O._sativa
	SC-1_I	O._sativa
	STONOR_I	Z._mays
	OSTONOR1_I	O._sativa
	OARE1_I	A._sativa
	BARE1_HV_I	H._vulgare
	WIS4_TM_I	T._monococcum
	ANGELA1_AT_I	A._tauschii
	RIRE1_I	O._australiensis
clade4	Copia1_HV_I	H._vulgare
	CPSC2_I	O._sativa
	CPSC4B_I	O._sativa
	SC-8_I	O._sativa
	SC-4_I	O._sativa
	ATCOPIA40_I	A._thaliana
	ATCOPIA65_I	A._thaliana
	ATCOPIA64_I	A._thaliana
	ATCOPIA63_I	A._thaliana

(continued)

Clades	Accession	Species
	ATCOPIA31_I	A._thaliana
	SHACOP8_I_MT	M._truncatula
	SHACOP21_I_MT	M._truncatula
	SHACOP18_I_MT	M._truncatula
	SHACOP4_I_MT	M._truncatula
	COP12_I_MT	M._truncatula
	COP20_I_MT	M._truncatula
	ATCOPIA78_I	A._thaliana
clade5	ATCOPIA43I	A._thaliana
	ENDOVIR1_I	A._thaliana
	COPIA3-I_LC	L._corniculatus
	SHACOP14_I_MT	M._truncatula
	INGA_HV_I	H._vulgare
	Copia3_HV_I	H._vulgare
	Maximus_I	H._vulgare
	SZ-37	O._sativa
	PREM2_ZM_I	Z._mays
	COPIA1-I_OS	O._sativa
	SZ-55_I	O._sativa
	COPIA-I_MT	M._truncatula
	COPIA2-I_LC	L._corniculatua
	TORTL1	L._esculentum
	COPIA4_I_MT	M._truncatula
	ATCOPIA71_I	A._thaliana
clade6	ATCOPIA72_I	A._thaliana
	ATCOPIA38_I	A._thaliana
	TOPSCOTCH_LP_I	L._pimpinellifolium
	COP21_I_MT	M._truncatula
	SHACOP6_I_MT	M._truncatula
	SHACOP7_I_MT	M._truncatula
	SHACOP12_I_MT	M._truncatula
	MTCOPIA1_I	M._truncatula
	SHACOP23_I_MT	M._truncatula
	COP10_I_MT	M._truncatula
	ATCOPIA75_I	A._thaliana
	ATCOPIA70_I	A._thaliana
	ATCOPIA13I	A._thaliana
clade7	TOS17	O._sativa
	SHACOP9_I_MT	M._truncatula
	LCCOPIA1_I	L._corniculatus
	TST1_I	S._tuberosum
	SHACOP11_I_MT	M._truncatula
	OSR3_I	O._sativa
	PDR1_I	P._sativum
clade8	SZ-6IN	O._sativa
	SZ-17_I	O._sativa
	OSCOPIA2_I	O._sativa

(continued)

Clades	Accession	Species
	COP_I_MT	M._truncatula
	COP3_I_MT	M._truncatula
	ATRE1_I	A._thaliana
	CASTOR_I	A._thaliana
	ATCOPIA6I	A._thaliana
	SHACOP3_I_MT	M._truncatula
	SHACOP20_I_MT	M._truncatula
	COP6_I_MT	M._truncatula
	Hopscotch_I	O._sativa
	RETROFIT_I	O._longistaminata
	COPIA1_ZM_I	Z._mays
	ATCOPIA82_I	A._thaliana
	ATCOPIA47_I	A._thaliana

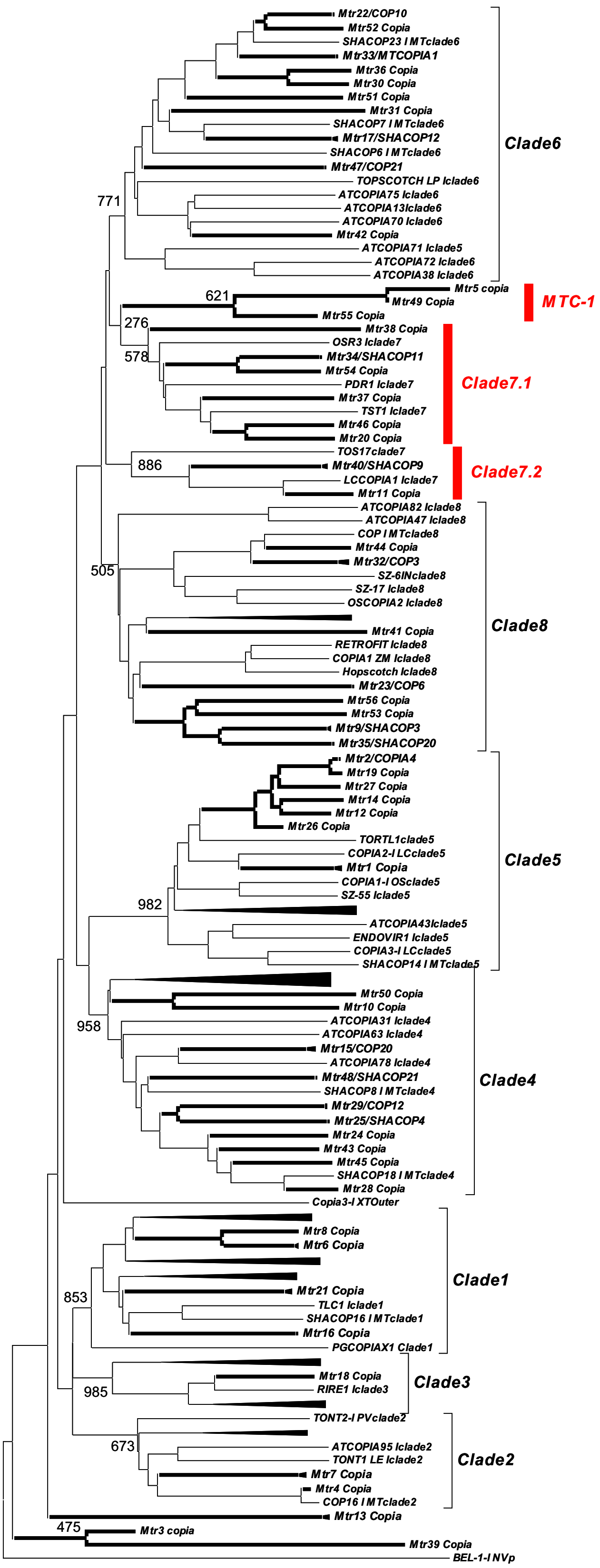

Figure S4.2: Tree of Copia superfamily. 1000 bootstraps have been performed. Only the stability of the 10 clades are shown. The labels of reference sequences are formatted as the element name directly followed by the clade id. Some lineages that do not contain families detected in this study are compressed.

References

Kordiš, D., 2005. A genomic perspective on the chromodomain-containing retrotransposons: Chromoviruses. *Gene*, **347**:161–173.

Lloréns, C., Futami, R., Bezemer, D., and Moya, A., 2008. The gypsy database (gydb) of mobile genetic elements. *Nucleic Acids Res*, **36**:D38–D46.

Macas, J. and Neumann, P., 2007. Ogre elements–a distinct group of plant ty3/gypsy-like retrotransposons. *Gene*, **390**:108–116.

Macas, J., Neumann, P., and Navrtilov, A., 2007. Repetitive dna in the pea (pisum sativum l.) genome: comprehensive characterization using 454 sequencing and comparison to soybean and medicago truncatula. *BMC Genomics*, **8**:427.
