# Supplementary figures and images for: LTR retrotransposon landscape in Medicago truncatula: more rapid removal than in rice
Source: BMC Genomics. 2008 Aug 10;9:382. doi: 10.1186/1471-2164-9-382 (PMC2533021; doi:10.1186/1471-2164-9-382)

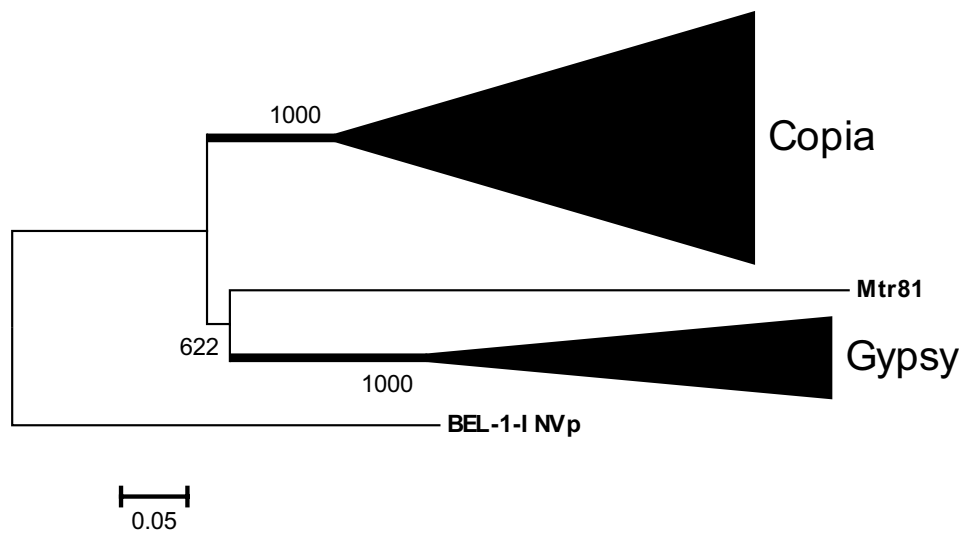

Figure S5.1: Phylogenetic position of Mtr81.

Supplement: Additional file 5 — Phylogenetic position of Mtr81. the RT sequence of Mtr81 do not belongs to Copia or Gypsy superfamily. It is placed as a third branch the phylogenetic tree. [file 1471-2164-9-382-S5.pdf]

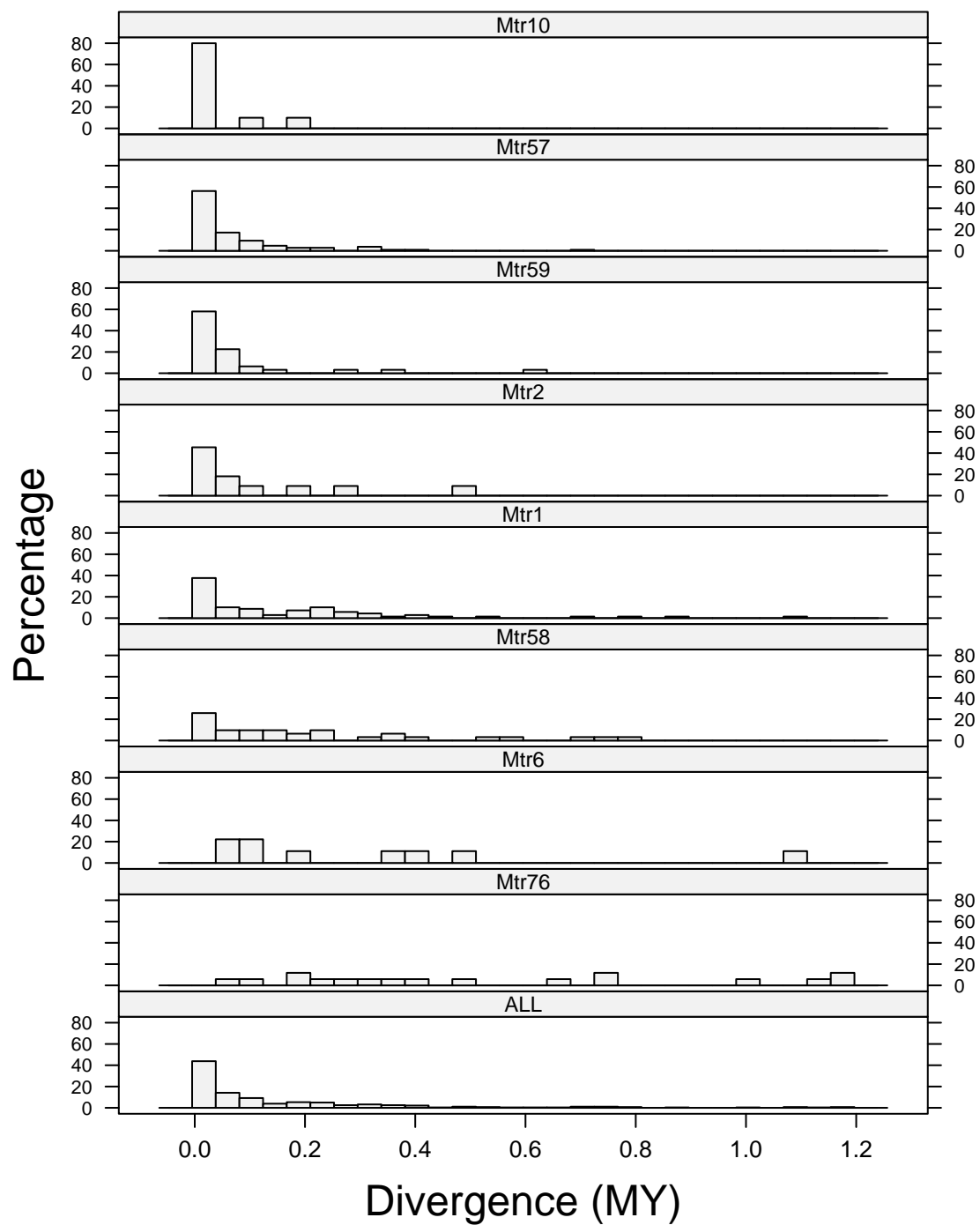

Figure S6.1: The insertion dates of 8 abundant LTR families

Supplement: Additional file 6 — Insertion dates of 8 abundant LTR families. this file contains a figure showing the distribution of the insertion time of 8 abundant LTR families. [file 1471-2164-9-382-S6.pdf]
